# Recurrent de novo *SPTLC2* variant causes childhood-onset amyotrophic lateral sclerosis (ALS) by excess sphingolipid synthesis

**DOI:** 10.1136/jnnp-2023-332132

**Published:** 2023-11-24

**Authors:** Safoora B Syeda, Museer A Lone, Payam Mohassel, Sandra Donkervoort, Pinki Munot, Marcondes C França, Juan Eli Galarza-Brito, Matthias Eckenweiler, Alexander Asamoah, Kenneth Gable, Anirban Majumdar, Anke Schumann, Sita D Gupta, Arpita Lakhotia, Perry B Shieh, A Reghan Foley, Kelly E Jackson, Katherine R Chao, Thomas L Winder, Francesco Catapano, Lucy Feng, Janbernd Kirschner, Francesco Muntoni, Teresa M Dunn, Thorsten Hornemann, Carsten G Bönnemann

**Affiliations:** 1 Neuromuscular and Neurogenetic Disorders of Childhood Section, National Institute of Neurological Disorders and Stroke, National Institutes of Health, Bethesda, MD, USA; 2 Institute of Clinical Chemistry, University Hospital Zürich, Zürich, Switzerland; 3 NIHR Great Ormond Street Hospital Biomedical Research Centre, London, UK; 4 Department of Neurology, University of Campinas, Campinas, Sao Paulo, Brazil; 5 Hospital Pablo Arturo Suarez, Quito, Ecuador; 6 Department of Neuropediatrics and Muscle Disorders, Faculty of Medicine, Medical Center, University of Freiburg, Freiburg, Germany; 7 Norton Children's Medical Group, University of Louisville School of Medicine, Louisville, KY, USA; 8 Department of Biochemistry and Molecular Biology, Uniformed Services University, Bethesda, Maryland, USA; 9 Department of Paediatric Neurology, Bristol Children's Hospital, Bristol, UK; 10 Department of Paediatrics and Adolescent Medicine, Faculty of Medicine, Medical Centre, University of Freiburg, Baden-Württemberg, Germany; 11 University of Louisville, Louisville, Kentucky, USA; 12 Department of Neurology and Pediatrics, University of California Los Angeles, Los Angeles, CA, USA; 13 Center for Mendelian Genomics, Program in Medical and Population Genetics, Broad Institute of MIT and Harvard, Cambridge, Massachusetts, USA; 14 Invitae Corporation, San Francisco, California, USA; 15 Dubowitz Neuromuscular Centre, CL Great Ormond Street Institute of Child Health and Great Ormond Street Hospital, London, UK; 16 Department of Biochemistry and Molecular Biology, Uniformed Services University of Health Sciences, Bethesda, MD, USA

**Keywords:** MOTOR NEURON DISEASE, ALS, NEUROGENETICS, NEUROMUSCULAR, BIOCHEMISTRY

## Abstract

**Background:**

Amyotrophic lateral sclerosis (ALS) is a neurodegenerative disease of the upper and lower motor neurons with varying ages of onset, progression and pathomechanisms. Monogenic childhood-onset ALS, although rare, forms an important subgroup of ALS. We recently reported specific *SPTLC1* variants resulting in sphingolipid overproduction as a cause for juvenile ALS. Here, we report six patients from six independent families with a recurrent, de novo, heterozygous variant in *SPTLC2* c.778G>A [p.Glu260Lys] manifesting with juvenile ALS.

**Methods:**

Clinical examination of the patients along with ancillary and genetic testing, followed by biochemical investigation of patients’ blood and fibroblasts, was performed.

**Results:**

All patients presented with early-childhood-onset progressive weakness, with signs and symptoms of upper and lower motor neuron degeneration in multiple myotomes, without sensory neuropathy. These findings were supported on ancillary testing including nerve conduction studies and electromyography, muscle biopsies and muscle ultrasound studies. Biochemical investigations in plasma and fibroblasts showed elevated levels of ceramides and unrestrained de novo sphingolipid synthesis. Our studies indicate that *SPTLC2* variant [c.778G>A, p.Glu260Lys] acts distinctly from hereditary sensory and autonomic neuropathy (HSAN)-causing *SPTLC2* variants by causing excess canonical sphingolipid biosynthesis, similar to the recently reported *SPTLC1* ALS associated pathogenic variants. Our studies also indicate that serine supplementation, which is a therapeutic in *SPTLC1* and *SPTCL2*-associated HSAN, is expected to exacerbate the excess sphingolipid synthesis in serine palmitoyltransferase (SPT)-associated ALS.

**Conclusions:**

*SPTLC2* is the second SPT-associated gene that underlies monogenic, juvenile ALS and further establishes alterations of sphingolipid metabolism in motor neuron disease pathogenesis. Our findings also have important therapeutic implications: serine supplementation must be avoided in SPT-associated ALS, as it is expected to drive pathogenesis further.

**WHAT IS ALREADY KNOWN ON THIS TOPIC**

- *SPTLC1* and *SPTLC2* variants are known to cause hereditary sensory and autonomic neuropathy (HSAN). We recently reported distinct *SPTLC1* variants cause juvenile amyotrophic lateral sclerosis (ALS), which is in striking contrast to the phenotype of HSAN. We also established excess sphingolipid synthesis as a new pathogenic mechanism for this form of juvenile ALS.

**WHAT THIS STUDY ADDS**

- Similar to the recently reported *SPTLC1* variant, we here report a recurrent de novo heterozygous *SPTLC2* variant causing juvenile ALS, which also acts distinctly from the HSAN-causing variants.

**HOW THIS STUDY MIGHT AFFECT RESEARCH, PRACTICE OR POLICY**

- *SPTLC2* is the second serine palmitoyltransferase-associated gene that underlies a form of monogenic, juvenile ALS and further establishes excess sphingolipid biosynthesis as a mechanism of motor neuron degeneration. This study also has therapeutic implications.

## Introduction

Amyotrophic lateral sclerosis (ALS) is a neurodegenerative disease of the upper and lower motor neurons with varying ages of onset, progression and with heterogeneous underlying pathomechanisms.^1^ Most ALS patients present in late adulthood with sporadic disease, while approximately 10% of patients have an underlying genetic aetiology.^2 3^ Although rare, familial and childhood-onset ALS are typically monogenic disorders and thus can provide important insights into the pathomechanism of motor neuron degeneration via distinct genetic, physiologic and metabolic alterations.^4^

Recently, we identified a monogenic, metabolic form of juvenile ALS, caused by specific variants in *SPTLC1* and established a novel mechanism linking these variants to excess sphingolipid biosynthesis and motor neuron degeneration.^5 6^ SPTLC1 and SPTLC2 are components of serine palmitoyltransferase (SPT), a multisubunit enzyme that catalyses the initial and rate-limiting step in sphingolipid biosynthesis by condensing L-serine and a fatty acyl-CoA (eg, palmitoyl-CoA) to form long-chain bases.^7^ In addition to SPTLC1 and SPTLC2, SPT complex normally includes one small subunit (ssSPT: SPTSSA or SPTSSB) that is an activator of the complex and ORMDL proteins that act as its natural inhibitors.^8 9^ *SPTLC1*-associated ALS variants disrupt SPT’s interaction with ORMDL proteins and thus result in overactivity of the enzyme complex.^5 10^

Alterations of SPT activity have been previously linked to neurodegeneration.^11 12^ Notably, pathogenic variants in *SPTLC1* (OMIM: 162400) and *SPTLC2* (OMIM: 613640) that cause a shift in SPT amino acid usage from L-serine to L-alanine or glycine result in elevated levels of 1-deoxysphingolipids (1-deoxySL) and clinically manifest with hereditary sensory autonomic neuropathy type 1 (HSAN1).^13–18^ This biochemical dichotomy, elevation of 1-deoxySL species versus excess canonical sphingolipid biosynthesis, underlies the divergent phenotypic manifestations of HSAN1^15^ and *SPTLC1* associated ALS, respectively.^5 10^ Further characterisation and understanding of pathogenic variants in the SPT complex that disrupt its normal function, their subsequent molecular and biochemical consequences, and how they lead to clinical manifestations of disease are essential for identifying therapeutic approaches that target this pathway in neurodegenerative diseases.

In this study, we report a second monogenic form of ALS linked to altered sphingolipid metabolism with six patients from independent families with a recurrent, de novo, dominantly acting, monoallelic pathogenic variant in *SPTLC2,* manifesting with severe juvenile ALS with rapid progression. We investigate and report the biochemical consequence of this variant on SPT complex activity, which, similar to ALS-associated *SPTLC1* variants, results in excess sphingolipid synthesis, in contradistinction to HSAN-associated *SPTLC1* and *SPTLC2* variants that result in SPT amino acid substrate shift and overproduction of 1-deoxySL.

## Methods

### Patient recruitment and sample collection

Patients were identified by their neurologist or geneticist. Medical history was obtained and clinical evaluations, including EMG/nerve conduction studies (NCS) and muscle biopsy, were performed as part of the standard diagnostic examination. Samples for research-based testing, including skin fibroblasts and blood were obtained using standard procedures. Muscle ultrasound was performed using Siemens 2000 using standard muscle ultrasound protocol and graded with Modified Heckmett scale.

### Genetic testing

Genetic testing performed in this cohort includes next generation-based sequencing, whole exome and whole genome. Confirmation of variants in individuals and parental segregation testing was performed by Sanger sequencing. The *SPTLC2* variant reported in this cohort was not present in gnomAD, Decipher, Leiden or TopMed databases.

### Sphingolipidomics of patient b samples

Lipids were extracted as described earlier.^10^ Briefly, lipids were extracted in a mixture of methanol: MTBE: chloroform 4:3:3 (v/v/v)) including a set of isotope labelled internal standards. After extraction, the single-phase supernatant was dried under N2 and stored at −20°C. For analysis, lipids were dissolved in MeOH and separated on a C30 LC column using Buffer (a) acetonitrile:water (6:4) with 10 mM ammonium acetate and 0.1% formic acid and (b) isopropanol:acetonitrile (9:1) with 10 mM ammonium acetate and 0.1% formic acid at a flow rate of 0.26 mL/minute. Eluting lipids are analysed on a Q-Exactive HRMS (ThermoFisher Scientific) in positive and negative mode using a heated electrospray ionisation. Identification was based on the predicted mass (resolution 5 ppm), the isotopic pattern, the retention time and specific fragmentation patterns. Pool samples at five concentrations were used as quality controls. Data analysis was performed with TraceFinder V.4.1 (Thermo Fisher Scientific) using single-point calibrations.

### Stable isotope labeling and sphingolipidomics of patient derived fibroblasts

Patient derived fibroblasts were isolated from skin biopsies. For sphingolipidomic studies, sample preparation and analysis were performed as previously described.^5^ In brief, cell pellets (~0.1–0.2 mg of protein) were mixed with internal sphingolipid standards (Avanti Polar Lipids, LM6002) in 1 mL of methanol, sonicated and mixed with 0.5 mL of chloroform and incubated overnight at 48°C. Insoluble material were then pelleted and removed. The samples were dried under nitrogen, dissolved in 0.3 mL of mobile phase A and B at 80:20 (v/v), sonicated and centrifuged before HPLC analysis. The HPLC mobile phase solvents used were (a) CH3OH:H2O:CH2O2 (74:25:1, v/v/v with 10 mM ammonium formate) and (b) CH3OH:CH2O2 (99:1, v/v with 10 mM ammonium formate). The samples were analysed using an Agilent 1200 Series HPLC coupled to an ABSciex QTRAP 4000 MS. The mass spectrometer was set to detect compounds in multiple reaction monitoring mode and compounds were quantified based on the ratio of the peak to the known concentration of the representative internal standard using the ABSciex Analyst programme as previously described.^19^

When deuterium-labelled amino acids (3,3-D2 L-Serine, DLM-161 or D4 L-alanine, Cambridge Isotope Laboratories) were used, it was added at the noted concentration 24 hours before harvesting the cells as follows: cells were trypsinised, washed with PBS and spun. The cell pellet was resuspended in PBS and divided into glass tubes for lipid extraction and Eppendorf tubes for protein quantification using the BCA assay (Thermo Fisher). Results for each sample was normalised to protein concentration. D2 labelled sphingomyelin (SM) measurements were corrected for the naturally prevalent+2 sphingomyelin species (~8.5%). Ceramide inhibition assay of SPT activity in patient fibroblasts was performed as described before.^5 10^

## Results

### Clinical features of patients with juvenile ALS

We identified six patients, from six independent families, presenting with a remarkably similar phenotype of childhood-onset progressive weakness. The weakness started in the lower extremities with subsequent involvement of the upper extremities, bulbar and respiratory muscles (table 1).

**Table 1:** Clinical phenotypic characteristics of *SPTLC2*-related ALS cohort

Patient	P1	P2	P3	P4	P5	P6
*SPTLC2* variant	c.778G>A; p.Glu260Lys	c.778G>A; p.Glu260Lys	c.778G>A; p.Glu260Lys	c.778G>A; p.Glu260Lys	c.778G>A; p.Glu260Lys	c.778G>A; p.Glu260Lys
Inheritance	de novo	de novo	de novo	de novo	de novo	de novo
Sex	M	F	F	F	M	F
Symptom onset (age)	congenital (in utero)	early childhood (18 months)	early childhood (2.5 years)	early childhood (4 years)	early childhood (2 years)	early childhood (3 years)
Age at last examination (years)	4	17	13	10	8	6
Age at walking/ambulation (age)	15 months	11 months	13 months	14 months	10 months	13 mo
Loss of independent ambulation (age)	3.5–4 years	10 years	7.5 years	ambulatory	4.5 years	4 years
Motor examination	Lower motor neuron findings:					
	Tongue fasciculations, proximal>distal weakness, generalised muscle atrophy	Tongue fasciculations, proximal>distal weakness, generalised muscle atrophy	Tongue and generalised fasciculations, proximal>distal weakness	Scalloped tongue with fasciculations, proximal>distal weakness	Tongue fasciculations, proximal>distal weakness, dropped head	Tongue fasciculation, proximal>distal weakness, dropped head
	Upper motor neuron findings:					
	Positive Babinski sign	Ankle clonus	LE spasticity, positive Babinski sign, ankle clonus	Pathologic hyper-reflexia	Pathologic hyper-reflexia, ankle clonus	Distal spasticity, pathologic hyper-reflexia, ankle clonus
Sensory examination	Normal	Normal	Normal	Normal	Normal	Normal
Respiratory involvement	Ventilation via tracheostomy (6 years)*	Nighttime NIV	NIV support (18 hours/day)	No	Nighttime NIV	No
Bulbar involvement	Dysphagia	Dysphagia	Dysphonia	No	Dysphagia	Dysphagia, dysphonia
Cognitive Involvement	Speech delay, autism	Word finding difficulties (8 years), decline in school performance (16 years). Memory loss, difficulties synthesising information. Pseudobulbar affect.	Decline in school performance (10 years), apathy and cognitive decline (13 years)	No	No	No
Sensory NCS	Normal	Normal	Normal	Normal	Normal	Normal
Motor NCS	Decreased CMAPs	Decreased CMAPs	Decreased CMAPs	Decreased CMAPs	Decreased CMAPs	Decreased CMAPs
Electromyography	Active denervation and chronic reinnervation changes	Active denervation and chronic reinnervation changes	Active denervation and chronic reinnervation changes	Active denervation and chronic reinnervation changes	Active denervation and chronic reinnervation changes	Active denervation and chronic reinnervation changes
Muscle biopsy (age)	Chronic neurogenic changes (3 years)	Chronic neurogenic changes (7 years)	NP	NP	Chronic neurogenic changes (7 years)	NP
MRI (age)	Normal brain MRI (3.5 years)	Normal brain and spine MRI (8 years, 14 years)	Normal brain and spine MRI (8 years, 10 years)	Normal brain and spine MRI (5 years)	Normal brain and spine MRI (5.5 years)	Normal brain and spine MRI (6 years)
Muscle ultrasound	Increased echogenicity with fasciculations	Increased echogenicity with fasciculations	NP	Increased echogenicity with fasciculations	NP	NP

*Died from a respiratory failure-related complication at age 7 years.

ALS, amyotrophic lateral sclerosis; CMAP, compound muscle action potential; F, female; LE, lower extremity; M, male; NCS, nerve conduction study; NIV, non-invasive ventilation; NP, not performed.

Onset of symptoms was in early childhood in the majority of the patients (n=5/6), with recognition of first symptoms ranging from 18 months to 4 years of age, except for patient P1 in whom the onset appears to be congenital with decreased movements in utero. Patients presented with initial toe walking and lower extremity spasticity, delay in gaining early motor milestones and frequent falls. All patients then had a rapid, progressive decline with regression in motor milestones, involvement of upper extremities and with eventual loss of ambulation in five patients thus far, which occurred as early as 4 years of age. Bulbar involvement (dysphagia and dysphonia) was noted in five patients. Progressive respiratory insufficiency has necessitated bilevel non-invasive ventilatory support or mechanical ventilation in half of the patients so far. One patient (P1) succumbed to a respiratory failure-related complication at 7 years of age. Two patients (P2 and P3) were also reported to show clear signs of cognitive decline with first symptoms manifesting at age 8 years in the form of word finding difficulties with progression in multiple cognitive modalities. Patient P2 also developed a pseudobulbar affect. One patient (P1) was reported to have speech delay and autistic features in early childhood. Family history was unremarkable in all patients.

**Figure 1:**
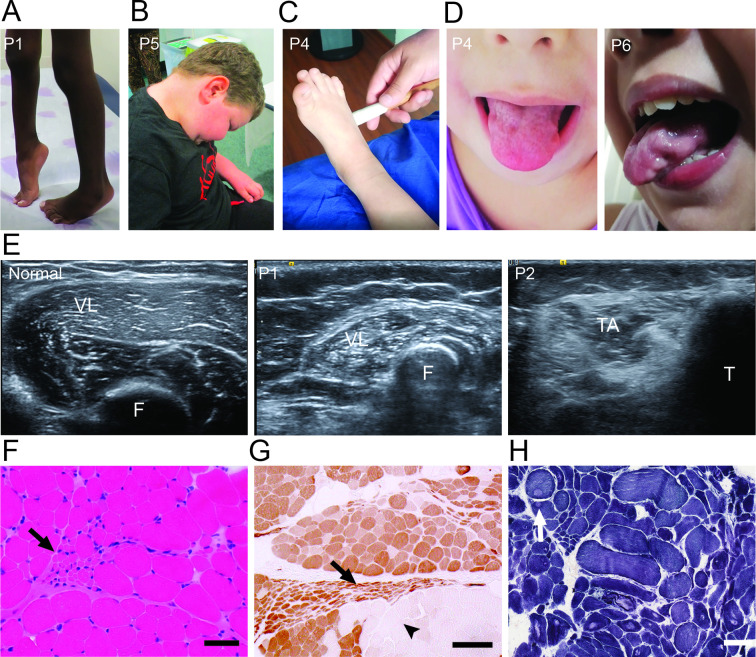
Clinical phenotypic characteristics of *SPTLC2* patients. (A) Patient P1 showing spasticity and toe walking. (B) P5 with significant axial (neck extension) weakness. (C) Patient P4 showing positive Babinski sign. (D) Patient P4 and P6 showing scalloped tongue (with active fasciculations on clinical examination). (E) Ultrasound of vastus lateralis (VL) muscle in an unaffected control (normal) compared with patient P1, which shows increased echogenicity with a characteristic ‘streaky’ pattern suggestive of neurogenic atrophy. Similar changes are noted in tibialis anterior muscle (TA) in patient P2. (F=Femur, T=Tibia). (F) Muscle biopsy of patient P1 stained with H&E and showing a group of atrophic muscle fibres (arrow). Scale bar=50 um. (G) ATPase 4.3 stain in P1 muscle biopsy shows severe chronic neurogenic changes with fibre type grouping and holofascicular atrophy (arrow). Note the adjacent fascicle with hypertrophic fibres (arrowhead). Scale bar=1—um. (H) Nicotinamide adenine dinucleotide stain in patient P5 showing targetoid fibres (arrow) indicative of neurogenic changes in muscle fibres.

On clinical examination, all patients showed a combination of upper and lower motor neuron signs with no sensory findings (figure 1A–D). Upper motor neuron involvement was evident in the form of spasticity, pathologic hyper-reflexia including an exaggerated jaw jerk, positive Babinski sign and/or ankle clonus. Fasciculations, including tongue fasciculations, proximal more than distal weakness and muscle atrophy, was seen in all patients, signifying multilevel lower motor neuron disease. Ancillary tests were also consistent with motor neuron disease. NCS performed in all of the patients showed normal sensory responses, normal conduction velocities and decreased amplitude in compound muscle action potentials. Electromyography revealed acute and chronic neurogenic changes with spontaneous activity in the form of fibrillation potentials, positive sharp waves and fasciculations as well as decreased recruitment of large amplitude and long duration motor unit action potentials in multiple myotomes with a non-length dependent pattern. Muscle ultrasound performed in three patients revealed muscle atrophy, increased echogenicity in a ‘streaky’ pattern, reflecting groups of atrophic fibres, in upper and lower extremities and spontaneous activity in the form of fasciculations, indicating neurogenic changes in the muscles (figure 1E, online supplemental video 1). Muscle biopsies were obtained in three patients revealing acute neurogenic changes such as angular myofiber atrophy, target fibres and chronic neurogenic changes such as fibre type grouping, pyknotic nuclear clumps and atrophic clusters which sometimes involved entire fascicles (figure 1F–H). P5 was found to have mild cardiomyopathy and was treated with a beta blocker. Brain MRI imaging was reported as normal in all patients (online supplemental figure 1A–C).

10.1136/jnnp-2023-332132.supp1 Supplementary data

10.1136/jnnp-2023-332132.supp2 Supplementary video

### Recurrent *SPTLC2* c.778G>A; p.Glu260Lys variant in juvenile ALS patients

**Figure 2:**
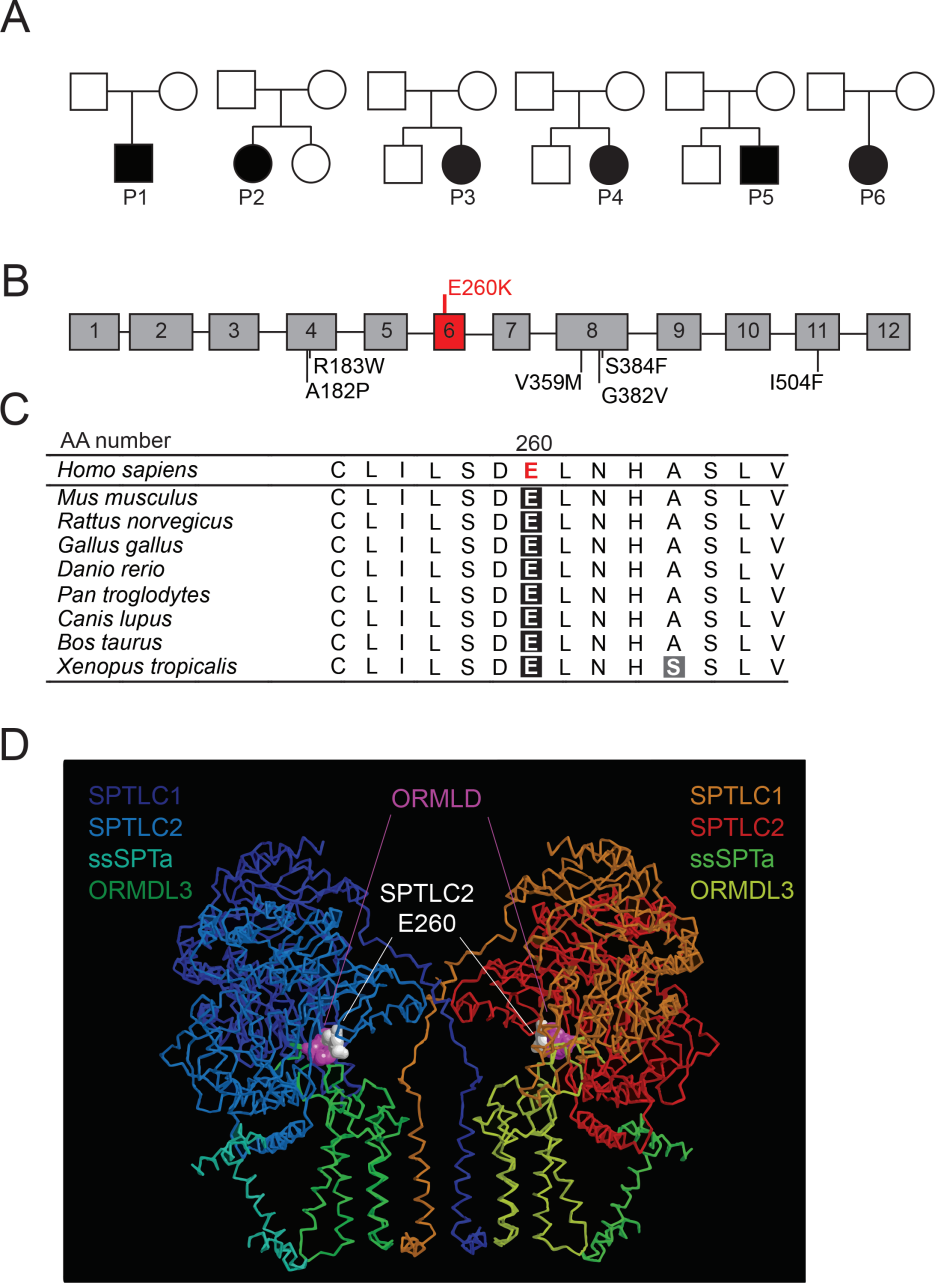
Pedigrees, schematic mapping of SPTLC2 E260K variant and in silico cryo-EM structure of SPT enzyme complex. (A) Pedigree of the families of six patients with de novo *SPTLC2* E260K variants. (B) Schematic of SPTLC2 and mapping of HSAN (black) and E260K (red) variants. (C) E260 is highly evolutionarily conserved across different species. (D) Cryo-EM structure of SPT enzyme complex showing its subunits, SPTLC1, SPTLC2 and ssSPTa in complex with ORMDL3. Note the direct contact of E260 residue of SPTLC2 with H7/S8 of ORMDL3 subunit. HSAN, hereditary sensory and autonomic neuropathy; SPT, serine palmitoyltransferase.

Next generation-based sequencing identified a recurrent heterozygous *SPTLC2* c.778G>A; [p.Glu260Lys] (GenBank: NM_004863.4) missense variant in all six patients. Parental segregation confirmed that the variant occurred de novo in all the patients (figure 2A,table 2). On standard relationship analysis, no discrepancies of paternity were noted. The p.E260K *SPTLC2* missense variant has not been reported to cause disease and is not present in the gnomADv3.1.2 database.^20^ It impacts an evolutionarily conserved Glutamine residue (figure 2B,C), and its change to Lysine is predicted to be deleterious based on various in silio models (Combined Annotation Dependent Depletion: 31, Polyphen: damaging, MutationTaster: disease causing). According to the variant classification criteria of the American College of Medical Genetics and Genomics the p.E260K *SPTLC2* variant is classified as likely pathogenic (PS2, PM2, PP3).^21^

**Table 2:** Genetic testing methodology and sequencing platforms

Patient	Methodology	Library preparation	Sequencing platform	Alignment/calling/annotation	Testing site
1	Trio WES	SeqCap EZ Exome+UTR Library (Roche)	TruSeq V2:39 Illumina	Seqr	NIH
2	Trio WGS	NA	Illumina	Illumina DRAGEN Bio-IT Platform v.2.03	PerkinElmer
3	Trio WES	Agilent SureSelect V.7 (Agilent)	Illumina HiSeq 2500	Varstation platform	UNICAMP
4	NGS Multigene pane	Custom IDT probes. (Integrated DNA Technologies)	Illumina Hi-Seq 2000	Novoalign (Novocraft Technologies, Selangor, Malaysia)/Genome Analysis Toolkit Unified Genotyper and Freebayes	Invitae
5	Trio WES	NA	Illumina	Twist Core Human Exome/Illumina	UK
6	Trio WES	Agilent SureSelectXt	Illumina R	Varvis (Limbus Technologies)	Commercial laboratory

NA, not available; NGS, next generation-based sequencing; v, version; WES, whole exome sequencing; WGS, whole genome sequencing.

### In silico mapping of SPTLC2 E260 based on the cryo-EM structure of SPT

We mapped our ALS associated *SPTLC2* variant on the recently reported cryoelectron microscopy structure of the SPT-ssSPT-ORMDL complex.^8 9^ This in silico mapping shows that the SPTLC2 E260 residue, which is mutated in our patients, is distinct from the enzyme active site, where most HSAN1 *SPTLC1* and *SPTLC2* variants cluster. In addition, SPTLC2 E260 makes direct contact with H7 of ORMDL3, suggesting its change could lead to impaired ORMDL3 regulation of SPT and loss of SPT inhibition (figure 2D). This prediction is mechanistically comparable with our previously reported loss of ORMDL-mediated inhibition of SPT in *SPTLC1-*associated ALS variants that cluster in the first transmembrane domain of SPTLC1, where it interacts with ORMDL3.^5 10^

### SL profile in *SPTLC2* E260K patient plasma samples

**Figure 3:**
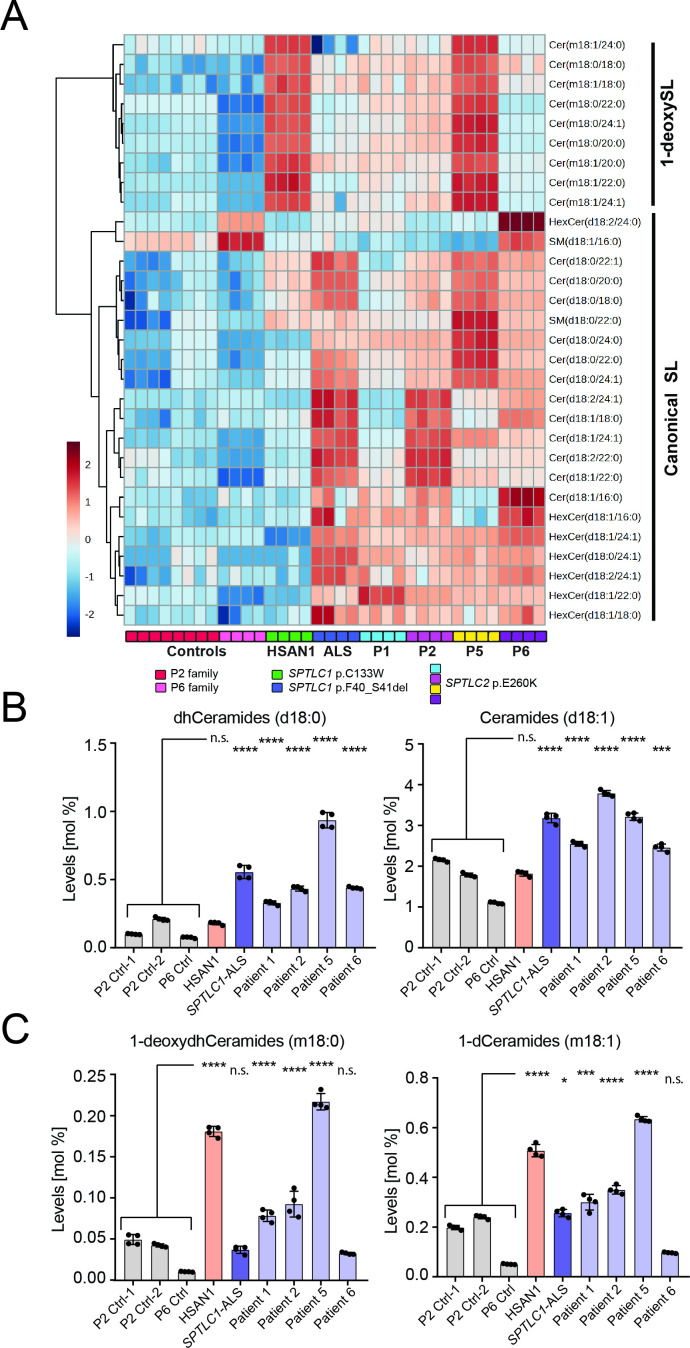
Sphingolipidomic profiling of E260K patient plasma. (A) Heat map cluster analysis of SL species from plasma of E260K patients compared with the controls and patients with *SPTLC1*-HSAN1 (C133W) and ALS (F40_S41del) variants. Absolute levels of each SL species were measured relative to internal lipid standard. Shown is the plot of the Log transformed (base 10) data with Pearson distance measure. Comparison of total plasma dihydroceramide (dhCeramide) and ceramides (B), and 1-deoxydihydroceramides (1-deoxydhceramide) and 1-deoxyceramides (C) from *SPTLC2* E260K patients, related controls, SPTLC1 HSAN1 (C133W) and SPTLC1-ALS (F40_S41del) patients. For bar graphs, data are represented as mean ± SD, n=4 independent replicates, one-way ANOVA with Dunnett adjustment was used for comparisons, * p<0.05, * p<0.05, *** p<0.001**** p<0.0001, n.s, not significant. ALS, amyotrophic lateral sclerosis.

We analysed the plasma SL profile in four patients with E260K *SPTLC2*- associated ALS (P1, P2, P5, P6) in comparison to three unaffected family controls. The SL profile of the p.E260K *SPTLC2* associated ALS patients showed a significant increase in plasma SL compared with controls (figure 3A,B). Similar to the previously reported ALS *SPTLC1* F40_S41del variant, the *SPTLC2* E260K variant was primarily associated with an increase in dihydroceramide, (dhCer; d18:0) and ceramide (Cer; 18:1) species (figure 3B). A principal component analysis (PCA) revealed a clustering of P1, P2 and P5, which was similar to the ALS SPTLC1 F40_S41del variant, but distinct from the controls (online supplemental figure 2A). Patient 6, but also the unaffected family 6 control sample, showed a distinct clustering indicating that the overall baseline SL profile in this family differs from that of the other patient and control samples (online supplemental figure 2A). A heatmap analysis revealed significant differences in the overall SL species profile between patients and controls (figure 3A). Relatively, dihydroceramide (dhCer) species (d18:0) were increased the most, but to a lesser extent, regular ceramides (d18:1 and d18:2) were also increased in the juvenile ALS patients. The heatmap signature of P1, P2, P5 and P6 was comparable to that of the previously reported ALS *SPTLC1* F40_S41del variant. However, sphingomyelin (SM), which is the most abundant SL in plasma and formed downstream of Cer, was not significantly increased, except for certain minor species, such as SM (d18:0/22:0) which appeared to be increased in ALS, but also HSAN1, plasma (figure 3A). In family 6, the profile differed in some specific SL species, such as HexCer (18:2/24:0) and SM (18:1/16:0), which is consistent with the different clustering of this family in the PCA.

In contrast, HSAN1 plasma (*SPTLC1* C133W) showed primarily an elevation in 1-deoxySL species but no change in canonical SLs. In most juvenile ALS patients, 1-deoxySL were not increased except for P5 who showed in addition to increased canonical SL also elevated plasma 1-deoxySL (figure 3C). In contrast to a previously described *SPTLC1* L39del ALS family,^10^ the increased 1-deoxySL in this patient seems not to be related to an altered alanine to serine ratio. The plasma amino acid profile was comparable to the other patients and family controls (online supplemental figure 2B). As all patients have the same underlying *SPTLC2* E260K variant, it is currently not fully explained why 1-deoxySL are elevated in P5.

**Figure 4:**
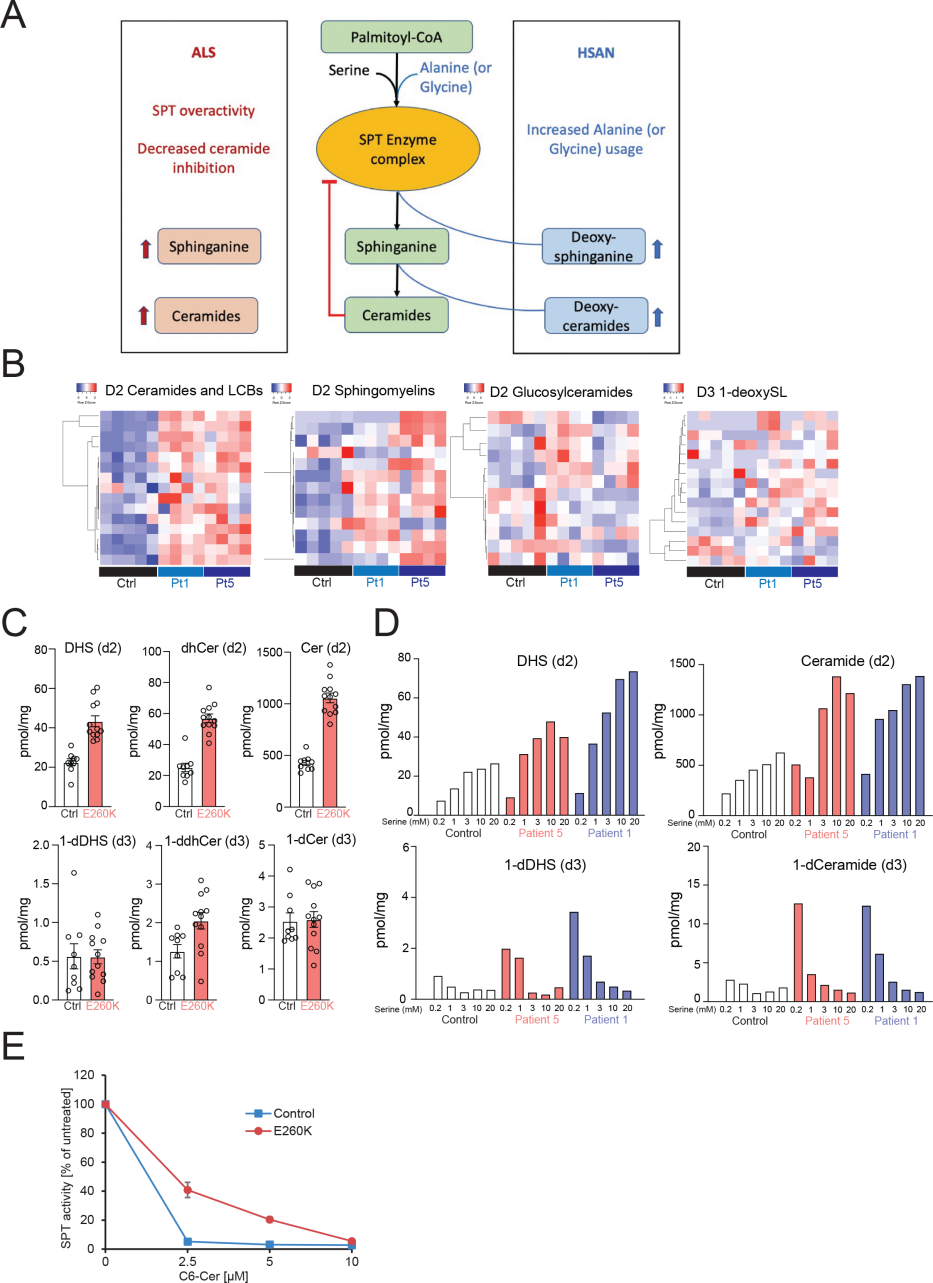
Spingolipidomic analysis showing increased synthesis of canonical sphingolipids in E260K patient fibroblast lines. (A) Serine palmitoyltransferase (SPT) catalyses the formation of sphingolipids by condensing serine and fattyacyl CoAs (typically palmitoyl CoA). SPTLC2 E260K variants, similar to SPTLC1-ALS variants, result in unrestrained production of canonical sphingolipids (sphinganine and ceramides) and decreased ORMDL/ceramide mediated inhibition. HSAN1-causing variants increase alanine (or glycine) usage by SPT leading to increased synthesis of 1-deoxysphingolipids. (B) Heatmap representation of individual sphingolipid species in *SPTLC2* ALS patient fibroblast lines (E260K) after stable radioisotope labelling with equimolar D2-serine and D4-alanine show increased production of canonical sphingolipids such as long chain bases (LCBs), ceramides, and to a lesser extent sphingomyelins and glucosyl ceramides. In contrast, there is no overproduction of 1-deoxysphingolipids (1-deoxySLs). (C) Total levels of de novo synthesised dihydrosphingosine (DHS), dihydroceramide (dhCer), Ceramide (Cer), 1-deoxyDHS (1-dDHS), 1-deoxydihydroceramide (1-ddhCer) and 1-deoxyceramide (1-dCer) in patient fibroblasts suggest a nearly twofold overproduction of canonical sphingolipids without notable overproduction of deoxySLs. Each data point represents a technical replicate of the control or patient fibroblasts from P1 and P5. (D) Addition of serine to the media exacerbates the overproduction of canonical sphingolipids, suggesting lack of homoeostatic negative feedback regulation of variant SPTLC2-containing SPT. Serine supplementation suppresses the 1-deoxySL production in E260K fibroblast lines, suggesting the overactivity is specific to canonical SL species. (E) SPT activity in control and patient derived fibroblasts after treatment with increasing doses of cell permeable C_6_-Ceramide (C_6_-Cer). The activities were recorded as total labelled SL produced relative to vehicle treated cells. SL de novo synthesis and activity measurements were performed in presence of D_3_,^15^N-L-Ser and D_4_-L-Ala. HSAN, hereditary sensory and autonomic neuropathy type 1.

Overall, the change in SL species observed for the *SPTLC2* E260K patients was comparable to the previously reported *SPTLC1* F40_41del variant and consistent with the concept that pathogenic variants in *SPTLC2* can result in impaired SL homoeostasis, and in increased de novo SL formation, as was recently shown for *SPTLC1* 5 6 10 (figure 4A).

### Overproduction of canonical sphingolipids in *SPTLC2* E260K patient derived fibroblasts

To study the biochemical consequences of the ALS-associated *SPTLC2* E260K variant on a cellular level, we used patient derived fibroblasts that reflect the disease-associated stoichiometry of mutant and wildtype SPTLC2 protein that is relevant in vivo. Patient derived fibroblasts were available from two patients (P2 and P5). We used stable isotope labelling with equimolar amounts of 3,3-D_2_ serine and D_4_-alanine followed by mass spectrometry and SL analysis to measure de novo rates of sphingolipid and 1-deoxySL synthesis in these cells. These experiments showed increased production of canonical sphingolipids including long chain bases, ceramides and to a lesser extent sphingomyelins and glucosylceramides as compared with the control samples (figure 4B,C). In contrast, there was no significant increase in production of 1-deoxySL. These findings in patient cells are consistent with the notion of unregulated canonical sphingolipid overproduction as the basis of SPT associated ALS as previously established by us for *SPTLC1*.^5^

### Serine supplementation and overproduction of sphingolipids in *SPTLC2* E260K patient derived fibroblasts

As serine supplementation can reduce 1-deoxySL production in SPTLC1 or SPTLC2-associated HSAN1, we tested the effect of serine supplementation at different doses in the patient derived fibroblast cultures. To monitor de novo synthesis rates, we again used stable isotope 3,3-D_2_ serine at varying concentrations and D_4_-alanine at 3 mM to monitor the de novo rates of canonical SL and 1-deoxySL synthesis, respectively. With increasing dose of serine, the canonical sphingolipids and ceramides were overproduced as compared with controls, while 1-deoxySL production rate decreased (figure 4D). These findings suggest that E260K SPTLC2 mutant SPT lacks normal homoeostatic regulation and overproduces canonical SLs, but not 1-deoxySLs.

### Ceramide inhibition of SPT activity in *SPTLC2* E260K patient derived fibroblasts

Ceramides are the main class of downstream SPT metabolites that exert inhibitory effects on its activity through ORMDL proteins.^22–24^ To further assess the homoeostatic regulation of mutant SPTLC2 containing SPT, we investigated the rate of SPT activity in response to increasing ceramide levels in patient derived fibroblasts. To verify whether the increased activity of the E260K variant is due to an impaired feedback regulation, we supplemented cells with membrane permeable C_6_-Ceramide (C_6_-Cer) to raise intracellular Cer levels.^24^ Ectopic addition of C_6_-Cer results in a dose dependent inhibition of SPT activity in both lines but showed a significant difference between wt and E260K fibroblasts (figure 4E). In wt cells, SPT activity was reduced by ~95% at 2.5 uM C_6_-Cer, but only by 60% in the mutant expressing cells.

These data show that the SPTLC2 E260K variant cause an impaired response to the inhibitory effects of ceramide, which is consistent with its impaired inhibition by ORMDLs.

## Discussion

Here, we characterise the phenotypic, genetic and biochemical profile of six patients from independent families with the identical de novo, heterozygous p.E260K variant in *SPTLC2,* presenting with early-childhood-onset weakness and spasticity. All patients had a combination of upper and lower motor neuron findings without sensory involvement and meet the El Escorial criteria for ALS.^25^ The clinical presentations across the cohort are strikingly similar, with early-childhood-onset spasticity, followed by rapidly progressive weakness involving upper and lower extremities, bulbar muscles, tongue fasciculations and eventual respiratory insufficiency, although with a variable rate of progression. Loss of ambulation occurred in five of the six patients, as early as 4 years of age. Although juvenile ALS is defined by onset of upper and lower motor neuron degeneration and related symptoms before 25 years of age, *SPTLC1* and *SPTLC2-*associated ALS together form a distinct, early childhood onset subgroup of neurodevelopmental motor neuron disease with typical onset of disease in the first few years of life. The majority of the patients report an early period of normal motor development preceding the onset of motor neuron disease. In addition, this ALS phenotype, presenting with upper and lower motor neuron involvement with normal sensory findings, is clinically distinct and contrasts the *SPTLC2-*associated HSAN1 phenotype, which is characterised by early onset of sensory loss, sensory neuropathy and variable autonomic involvement, and in later stages, mild motor neuropathy.^13 14^

The single heterozygous de novo *SPTLC2* E260K variant is absent from the population databases, changes a highly conserved residue, and is predicted to be deleterious by various in-silico prediction models. Our biochemical studies of patient serum and cellular models confirm its pathogenicity and probable biochemical pathomechanism. Consistent with our previous findings for *SPTLC1,* 5 6 10 the phenotypic distinction between HSAN1 and ALS causing *SPTLC2* variants correspond to distinct biochemical profiles. In *SPTLC2* associated HSAN1, there is increased synthesis of 1-deoxySL.^18^ In contrast, despite some variability, overproduction of canonical sphingolipids and in particular ceramides was notable in the ALS causing E260K *SPTLC2* variant in all samples analysed. Although one of the patients (P5) also showed increased 1-deoxySL species in serum, patient derived fibroblasts including those of P5 did not show increased de novo synthesis of 1-deoxySL. In addition, there was no evidence of sensory involvement clinically or on electrodiagnostic studies and thus the clinical relevance of this finding in this patient remains unclear. Serum levels of 1-deoxySL can be influenced by systemic lipoprotein and amino acid metabolic changes,^10^ and thus may not reflect their levels in other tissues, including sensory and motor neurons, which are protected by the blood brain and nerve barrier. In serum samples, biochemical variability is also reflected in the metabolic profile of family of P6, which while consistent with the ALS associated SL profile, clusters separately for some of the SL species, suggesting again genetic or metabolic modifiers. The cellular metabolic consequences of pathogenic variants in *SPTLC1* and *SPTLC2* are therefore better represented by studies in cellular systems, as ALS, HSAN1 and overlap biochemical phenotypes (eg, in the *SPTLC1* S331 variants)^26^ are clearly distinguished in cells.^10^ By administering equimolar amounts of serine and alanine in culture media, these studies more directly assess the SPT enzyme’s substrate preference. Our analysis of de novo sphingolipid synthesis of mutant SPTLC2 in patient derived fibroblasts were consistent with the biochemical mechanism previously reported in *SPTLC1*-associated ALS patients. Similar to *SPTLC1* ALS variants, this *SPTLC2* ALS variant also results in overproduction of canonical sphingolipids in patient derived fibroblasts without increasing 1-deoxySL production rates (figure 4B,C). In patient plasma, we also saw an increase in SL species with an uncommon N-acyl chain (C_20:0_, C_22:0_ and C_22:1_) which are normally minor or absent in plasma (figure 3A). This is consistent with the pattern we observed earlier in SPTLC1-ALS patient plasma and SPTLC1-ALS mutant expressing cells.^5 6 10^ As the conjugation of the N-acyl chain happens downstream of SPT, these differences might be secondarily related to the increased SPT activity and increased flux through the pathway.

*SPTLC1*-related and *SPTLC2*-related HSAN1 variants result in overproduction of 1-deoxySL species by increasing L-alanine (or glycine) usage instead of L-serine by the SPT complex.^27^ Thus, in HSAN1, L-serine supplementation has the potential to serve as an effective therapeutic by increasing serine usage by the SPT complex and by ameliorating the underlying biochemical abnormality.^28^ In case of an overactive SPT complex, however, L-serine supplementation would stimulate SL formation even further and is predicted to worsen sphingolipid overproduction. Indeed, when we tested the effect of increasing exogenous serine on de novo rates of sphingolipid synthesis by SPT in patient derived fibroblasts, we noted a further increase in canonical sphingolipid overproduction, consistent with a homoeostatically unregulated SPT. As we have already shown for *SPTLC1* related ALS^5^, this notion has important therapeutic implication in our *SPTLC2*-ALS patients also, arguing against serine supplementation in *SPTLC2*-ALS which may drive the pathogenesis further.

Similar to the mechanism we reported previously for the *SPTLC1* ALS variants,^5^ we predict that this overproduction of sphingolipids in the E260K *SPTLC2* variant is secondary to an impaired inhibitory effect of ORMDLs on the SPT complex. The increased SL synthesis might result from an impaired feedback inhibition as the response to externally added C_6_Cer was reduced. However, we cannot fully exclude that the E260K variant also has an impact on the general catalytic activity of the enzyme.

While we plan to study the effect of the E260K variant on ORMDL inhibition further, in silico modelling of the SPTLC2 E260 residue on the cryo-EM structure of SPT suggests that this residue interacts with ORMDL3 H7 residue, in a highly dynamic part of the enzyme complex. This observation in conjunction with an analogous mechanism of loss of ORMDL regulation in *SPTLC1* associated ALS provides a structural explanation for loss of ORMDL inhibition of SPT as a common mechanism for neurodegeneration that clinically manifests as early-onset ALS.

While there are striking clinical and metabolic similarities between SPTLC1 and SPTLC2 associated ALS phenotypes, based on the currently known cases there are also interesting potential differences emerging. Progression in E260K *SPTLC2* associated ALS is quite rapid with respiratory involvement and loss of ambulation within a few years of onset as compared with the somewhat more slowly progressive *SPTLC1* ALS. Notably, and in contrast with *SPTLC1-*associated ALS patients, progressive cognitive decline was notable in two patients (P2 and P3), and one patient (P1) had speech delay and autistic features suggestive of a broader neurodegenerative process. Although brain MRI imaging obtained early in the course of the disease, before recognition of cognitive involvement, was unremarkable (online supplemental figure 1), we suspect that the observed cognitive findings in some of the patients are part of the same neurodegenerative process that initially involved upper and lower motor neurons. Recognition of ALS, including its sporadic forms, as a broader neurodegenerative disease with a predilection for frontal and temporal lobes is thus consistent with our findings in *SPTLC2* E260K ALS.

It is notable that HSAN1 patients may also develop central nervous system involvement in the form of macular telangiectasias and subclinical findings of hyper-reflexia on examination.^29^ However, a broad and progressive neurodegenerative disease is not a typical feature of HSAN1. Reduced serum levels of serine and increased 1-deoxySL have been previously noted in patients with macular telangiectasia type 2 (with or without associated SPT component pathogenic variants).^30^ However, our patients with E260K SPTLC2 related ALS phenotype did not show any clinical signs of macular involvement at the time of evaluation.

Several questions remain to be answered. SLs are ubiquitous in all cells, but it remains unclear as to why their increased formation is associated with degeneration of motor neurons without involvement of other neuronal and non-neuronal tissues. It might be that in SPT-associated ALS, a certain subset of neurotoxic sphingolipids are formed that lead to neurodegeneration. However, additional functional and biochemical studies in appropriate cellular and animal models will be needed to study this association further. In addition, additional disease-causing variants may emerge for *SPTLC2,* further broadening the associated phenotypes and pathophysiological insights(table 2).

## Data Availability

All data relevant to the study are included in the article or uploaded as online as supplemental information.

## References

[1] Al-Chalabi A , Hardiman O . The epidemiology of ALS: a conspiracy of genes, environment and time. Nat Rev Neurol 2013;9:617–28. 10.1038/nrneurol.2013.203 24126629

[2] Mejzini R , Flynn LL , Pitout IL , et al . ALS genetics, mechanisms, and therapeutics: where are we now Front Neurosci 2019;13:1310. 10.3389/fnins.2019.01310 31866818 PMC6909825

[3] Alsultan AA , Waller R , Heath PR , et al . The genetics of amyotrophic lateral sclerosis: current insights. Degener Neurol Neuromuscul Dis 2016;6:49–64. 10.2147/DNND.S84956 30050368 PMC6053097

[4] Lehky T , Grunseich C . Juvenile amyotrophic lateral sclerosis: a review. Genes (Basel) 2021;12:1935. 10.3390/genes12121935 34946884 PMC8701111

[5] Mohassel P , Donkervoort S , Lone MA , et al . Childhood amyotrophic lateral sclerosis caused by excess sphingolipid synthesis. Nat Med 2021;27:1197–204. 10.1038/s41591-021-01346-1 34059824 PMC9309980

[6] Lone MA , Zeng S , Bourquin F , et al . SPTLC1 p.Leu38Arg, a novel mutation associated with childhood ALS. Biochim Biophys Acta Mol Cell Biol Lipids 2023;1868:159359. 10.1016/j.bbalip.2023.159359 37348646

[7] Harrison PJ , Dunn TM , Campopiano DJ . Sphingolipid biosynthesis in man and microbes. Nat Prod Rep 2018;35:921–54. 10.1039/c8np00019k 29863195 PMC6148460

[8] Li S , Xie T , Liu P , et al . Structural insights into the assembly and substrate selectivity of human SPT-ORMDL3 complex. Nat Struct Mol Biol 2021;28:249–57. 10.1038/s41594-020-00553-7 33558762

[9] Wang Y , Niu Y , Zhang Z , et al . Structural insights into the regulation of human serine palmitoyltransferase complexes. Nat Struct Mol Biol 2021;28:240–8. 10.1038/s41594-020-00551-9 33558761 PMC9812531

[10] Lone MA , Aaltonen MJ , Zidell A , et al . SPTLC1 variants associated with ALS produce distinct sphingolipid signatures through impaired interaction with ORMDL proteins. J Clin Invest 2022;132:e161908. 10.1172/JCI161908 35900868 PMC9479574

[11] Zhao L , Spassieva S , Gable K , et al . Elevation of 20-carbon long chain bases due to a mutation in serine palmitoyltransferase small subunit B results in neurodegeneration. Proc Natl Acad Sci U S A 2015;112:12962–7. 10.1073/pnas.1516733112 26438849 PMC4620873

[12] Wang G , Bieberich E . Sphingolipids in neurodegeneration (with focus on ceramide and S1P). Adv Biol Regul 2018;70:51–64. 10.1016/j.jbior.2018.09.013 30287225 PMC6251739

[13] Fridman V , Oaklander AL , David WS , et al . Natural history and biomarkers in hereditary sensory neuropathy type 1. Muscle Nerve 2015;51:489–95. 10.1002/mus.24336 25042817 PMC4484799

[14] Houlden H , King R , Blake J , et al . Clinical, pathological and genetic characterization of hereditary sensory and autonomic neuropathy type 1 (HSAN I). Brain 2006;129:411–25. 10.1093/brain/awh712 16364956

[15] Penno A , Reilly MM , Houlden H , et al . Hereditary sensory neuropathy type 1 is caused by the accumulation of two neurotoxic sphingolipids. J Biol Chem 2010;285:11178–87. 10.1074/jbc.M109.092973 20097765 PMC2856995

[16] Dawkins JL , Hulme DJ , Brahmbhatt SB , et al . Mutations in SPTLC1, encoding serine palmitoyltransferase, long chain base Subunit-1, cause hereditary sensory neuropathy type I. Nat Genet 2001;27:309–12. 10.1038/85879 11242114

[17] Bejaoui K , Wu C , Scheffler MD , et al . SPTLC1 is mutated in hereditary sensory neuropathy, type 1. Nat Genet 2001;27:261–2. 10.1038/85817 11242106

[18] Rotthier A , Auer-Grumbach M , Janssens K , et al . Mutations in the SPTLC2 subunit of serine palmitoyltransferase cause hereditary sensory and autonomic neuropathy type I. Am J Hum Genet 2010;87:513–22. 10.1016/j.ajhg.2010.09.010 20920666 PMC2948807

[19] Merrill AH , Sullards MC , Allegood JC , et al . Sphingolipidomics: high-throughput, structure-specific, and quantitative analysis of sphingolipids by liquid chromatography tandem mass spectrometry. Methods 2005;36:207–24. 10.1016/j.ymeth.2005.01.009 15894491

[20] Karczewski KJ , Francioli LC , Tiao G , et al . The mutational constraint spectrum quantified from variation in 141,456 humans. Nature 2020;581:434–43. 10.1038/s41586-020-2308-7 32461654 PMC7334197

[21] Richards S , Aziz N , Bale S , et al . Standards and guidelines for the interpretation of sequence variants: a joint consensus recommendation of the American college of medical genetics and genomics and the association for molecular pathology. Genet Med 2015;17:405–24. 10.1038/gim.2015.30 25741868 PMC4544753

[22] Davis DL , Gable K , Suemitsu J , et al . The ORMDL/Orm-serine palmitoyltransferase (SPT) complex is directly regulated by ceramide: reconstitution of SPT regulation in isolated membranes. J Biol Chem 2019;294:5146–56. 10.1074/jbc.RA118.007291 30700557 PMC6442065

[23] Hjelmqvist L , Tuson M , Marfany G , et al . ORMDL proteins are a conserved new family of endoplasmic reticulum membrane proteins. Genome Biol 2002;3:RESEARCH0027. 10.1186/gb-2002-3-6-research0027 12093374 PMC116724

[24] Siow DL , Wattenberg BW . Mammalian ORMDL proteins mediate the feedback response in ceramide biosynthesis. J Biol Chem 2012;287:40198–204. 10.1074/jbc.C112.404012 23066021 PMC3504734

[25] Brooks BR , Miller RG , Swash M , et al . El escorial revisited: revised criteria for the diagnosis of amyotrophic lateral sclerosis. Amyotroph Lateral Scler Other Motor Neuron Disord 2000;1:293–9. 10.1080/146608200300079536 11464847

[26] Fiorillo C , Capodivento G , Geroldi A , et al . The SPTLC1P. S331 mutation bridges sensory neuropathy and motor neuron disease and has implications for treatment. Neuropathol Appl Neurobiol 2022;48:e12842. 10.1111/nan.12842 35904184 PMC9804203

[27] Lone MA , Santos T , Alecu I , et al . 1-Deoxysphingolipids. Biochim Biophys Acta Mol Cell Biol Lipids 2019;1864:512–21. 10.1016/j.bbalip.2018.12.013 30625374

[28] Eichler FS , Hornemann T , McCampbell A , et al . Overexpression of the wild-type SPT1 subunit lowers desoxysphingolipid levels and rescues the phenotype of HSAN1. J Neurosci 2009;29:14646–51. 10.1523/JNEUROSCI.2536-09.2009 19923297 PMC3849752

[29] Triplett J , Nicholson G , Sue C , et al . Hereditary sensory and autonomic neuropathy type IC accompanied by upper motor neuron abnormalities and type II Juxtafoveal retinal Telangiectasias. J Peripher Nerv Syst 2019;24:224–9. 10.1111/jns.12315 30866134

[30] Gantner ML , Eade K , Wallace M , et al . Serine and lipid metabolism in macular disease and peripheral neuropathy. N Engl J Med 2019;381:1422–33. 10.1056/NEJMoa1815111 31509666 PMC7685488

