# Supplementary material for: Recurrent de novo SPTLC2 variant causes childhood-onset amyotrophic lateral sclerosis (ALS) by excess sphingolipid synthesis
Source: J Neurol Neurosurg Psychiatry. 2023 Nov 24;95(2):103–13. doi: 10.1136/jnnp-2023-332132 (PMC10850718; doi:10.1136/jnnp-2023-332132)
Supplement: Supplementary data [file jnnp-2023-332132supp001.pdf]

Supplementary methods

Amino acid analysis

Amino acids were extracted from 10 µL plasma precipitated with 180 µl ice-cold methanol containing 1 nmol of stable isotope labelled amino acids (Cambridge Isotope Laboratories, MSK-A2-1.2). Samples were incubated at -20°C for 30 min followed by centrifugation at 4°C (14,000g, 10 min). The supernatant was transferred to a fresh tube and dried under a N2 stream and stored at -20°C until analysis. Dried pellets were re-constituted in 100 µL of 0.1 % acetic acid and separated on a reverse-phase C18 column (EC 250/2 NUCLEOSIL 100-3 C18HD, L=250 mm, ID: 2 mm; Macherey-Nagel). 5 µL were subjected to liquid chromatography coupled with multiple reaction monitoring (MRM) mass spectrometry using a QTRAP 6500+ LC-MS/MS-MS System (Sciex). Solvent systems used were (A) 0.1 % formic acid in water and (B) acetonitrile (100 %) at a flow rate of 0.2 ml/min. Sample ionization was achieved via electrospray ionization in positive ion mode. Quantification was performed using MultiQuant (2.1) software (SCIEX).

Supplementary figures and video:

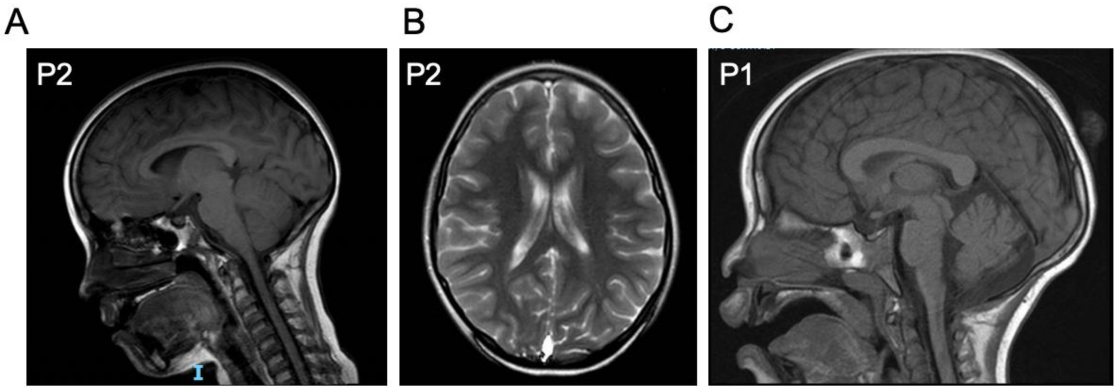

Supplementary Figure 1: **Brain MRI images of patients with *SPTLC2* E260K variant. A**. T1 sagittal brain MRI images of P2 obtained at 8 years of age shows normal brain parenchyma and structure. **B.** Representative Axial T2 image of brain MRI in P2 is also unremarkable. Myelination pattern is normal. **C.** T1 sagittal brain MRI image of P1 obtained at 3 years and 8 months of age is unremarkable.

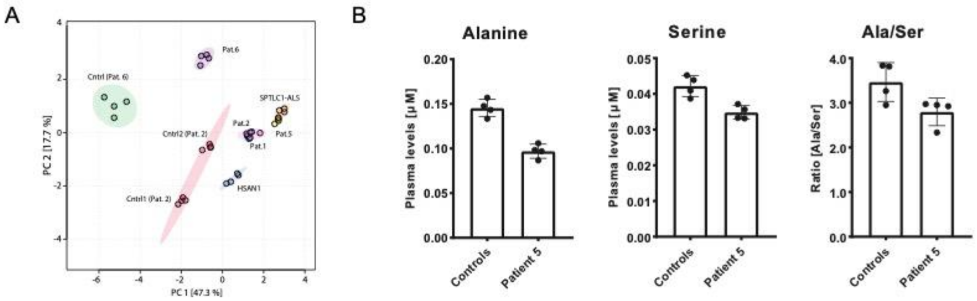

Supplementary Figure 2: Principal component analysis, SPT substrate amino acid levels and ceramide inhibition assay. **A**. Principal component analysis (PCA) and comparison of plasma ceramides and 1-deoxyceramides from *SPTLC2* E260K patients, relative to unaffected family member controls, SPTLC1 HSAN1 (C133W), and SPTLC1-ALS (F40S41del) patients. **B**. Absolute and relative levels of SPT substrate amino acids, Ser and Ala from pE260K carrier patient 5 and controls (n=4).

Supplementary video 1: **Muscle ultrasound showing active fasciculations in right vastus lateralis of patient P1**

(Video attached seperately)
